# TRPV2: a universal regulator in cellular physiology with a yet poorly defined thermosensitivity

**DOI:** 10.1186/s12576-024-00936-1

**Published:** 2024-09-16

**Authors:** Tabea C. Fricke, Andreas Leffler

**Affiliations:** Department of Anesthesiology and Intensive Care Medicine, Hannover Medical School, Carl-Neuberg Strasse 1, 30625 Hannover, Germany

**Keywords:** Ion channel, Thermosensitivity, Heat, TRPV2, Physiology

## Abstract

Transient receptor potential (TRP) ion channels serve as sensors for variations in ambient temperature, modulating both thermoregulation and temperature responsive cellular processes. Among these, the vanilloid TRP subfamily (TRPV) comprises six members and at least four of these members (TRPV1-TRPV4) have been associated with thermal sensation. TRPV2 has been described as a sensor for noxious heat, but subsequent studies have unveiled a more complex role for TRPV2 beyond temperature perception. This comprehensive review aims to elucidate the intricate thermosensitivity of TRPV2 by synthesizing current knowledge on its biophysical properties, expression pattern and known physiological functions associated with thermosensation.

## Background

Mammals and other animals expend significant energy to maintain a constant body temperature, regardless of environmental conditions. This process, known as *thermal regulation*, relies on complex mechanisms. Initially, the body and ambient temperatures need to be assessed. External receptor cells, primarily located in the skin, detect environmental temperature changes, while internal receptors within various organs sense body temperature.

Transient receptor potential (TRP) ion channels play pivotal roles in organisms, enabling the regulation of body temperature and the detection as well as response to fluctuations in the surrounding temperature. Within thermosensitive sensory neurons, a subset of TRP channels are expressed that exhibit distinct temperature thresholds for activation by either heat or cold [1]. The initial identification and functional characterization of the recombinant rat TRPV2 orthologue implicated a role as a heat sensor activated by high temperatures exceeding 50 °C [2]. Due to a strong expression in thinly myelinated sensory neurons, a role of TPRV2 in high threshold type 1 mechano-sensitive and heat-sensitive Aδ-fibers in rodents and humans was proposed [2–4]. However, the yet only report on mice with a global genetic deletion of TRPV2 demonstrated an intact heat sensitivity in various pain models [5]. Given that the human TRPV2 orthologue even appears to be insensitive to heat, it seems like evolutionary pressure did not maintain the thermosensitive property of TRPV2 [6]. Furthermore, the role of TRPV2 in non-neuronal tissues including immune cells and different tumors does not seem to require thermosensitivity. Consequently, scientific efforts to further explore the heat-sensitivity of TRPV2 have been limited and, therefore, its physiological meaning remains uncertain.

### Thermosensitivity of TRPV2 in in vitro cellular systems

The mammalian TRP superfamily comprises 28 members classified into six major subfamilies: TRPA (ankyrin), TRPC (canonical), TRPP (polycystin), TRPV (vanilloid), TRPM (melastatin), and TRPML (mucolipin). Among these subfamilies, TRPV [1–4], TRPA1, TRPM [2, 3, 8], and TRPC [5] channels have been described as key players in thermosensation [7–10]. To determine whether a protein is a thermosensor or sensitive to temperature changes, it is quantified by the “*Q*_*10*_* value*”, a unit less measure indicating the ratio of a property assessed at two temperatures differing by 10 °C [11]. In general, processes with Q_10_ values below ~ 4 are typically deemed less temperature-sensitive, whereas those with Q_10_ values exceeding ~ 7 are regarded as highly thermosensitive [11]. The *Q*_10_-values of for each channel have been documented to exhibit significant variation. For instance, the values for TRPV1, TRPV3, and TRPV4 channels are 40, 22, and 10–19, respectively, thus within the thermosensitive range [12–15]. TRPV2 has been reported to have a *Q*_10_-value > 100, indicating that it is a highly thermosensitive ion channel [16].

A growing number of reports have demonstrated that heterologously expressed recombinant rat TRPV2 exhibit heat-evoked membrane currents with a threshold > 50 °C. Recombinant mouse TRPV2 channels have not been examined in detail, but they can also be activated by heat [17]. More important is the notion that the human TRPV2 orthologue seems to be completely insensitive to heat up to 60 °C [6, 17]. This clear species-specific difference between rodent and human TRPV2 orthologues may imply that the physiological relevance of the thermosensitivity of TRPV2 may be limited and has not been conserved by evolutionary pressure. As the thermosensitivities of TRPV2 orthologues from other species have not yet been investigated, this notion remains speculative and should be further explored. All in all, relatively little is known about both properties of heat-evoked membrane currents generated by TRPV2. Even less is known about the structural determinants encoding for the heat sensitivity of rodent TRPV2, as for the species-specific heat-sensitivity. Caterina and colleagues initially described that recombinant rat TRPV2 channels generate heat-evoked membrane currents with a high threshold > 52 °C when expressed in oocytes or HEK 293 cells [2]. They also demonstrated that heat-evoked currents through TRPV2 are non-selective for cations with a high permeability for calcium, and that these currents undergo a prominent use-dependency upon repetitive stimulation, e.g., the high threshold for activation decreases below 40 °C [2]. In more recent reports, we and other laboratories demonstrated that chemical agonists of TRPV2 including oxidants, UVA-light, weak acids, probenecid and cannabinoids can sensitize TRPV2 to heat and thus strongly reduce the high threshold for activation [17–19]. A latest report also demonstrated that phosphorylation of intracellular tyrosine residues can robustly sensitize rat TRPV2 to heat as well [20]. Besides the finding that human TRPV2 also generated small heat-evoked currents following oxidation, it is not known if other agonists can render human TRPV2 heat sensitive [17].

To identify the molecular determinants encoding for the species-specific differences between rodent and human TRPV2, Neeper et al. used calcium imaging experiments to examine chimeric constructs with swapped intracellular N-terminal or C-terminal domains as well as the six-trans domain with the pore region [6]. However, none of the investigated chimeric channels was activated by heat. The authors also designed several deletion mutations of the N- and C-termini of rat TRPV2 and examined their responses to 2-APB and heat. The channel lost functionality when 83 or more amino acids were deleted from the N-terminus, and also when 55 amino acids were deleted from the C-terminus. The authors concluded that both termini are essential for the functionality of the channel, but effectively the study failed to determine which regions of the TRPV2 channel are responsible for heat-sensitivity [6]. Technically more elaborated patch clamp studies from the Qin-laboratory provided more detailed insights into possible mechanisms in rat and human TRPV2 [21–23]. In a report from 2011, Yao and colleagues examined chimeric channels of rat TRPV1 and TRPV2 to identify regions that determine their different temperature dependencies [21]. The key finding for TRPV2 in this study was that the transfer of the proximal half of the N-terminal region (MDP “membrane-proximal domain” located between the ankyrin repeat domain and the transmembrane domain) from TRPV1 to TRPV2 resulted in a significantly altered temperature sensitivity such that the threshold of TRPV2-construct was strongly reduced [21]. The authors also applied the same strategy to human TRPV2, e.g., they swapped the MPD from rat TRPV1 into the heat-insensitive human TRPV2. This chimeric channel indeed generated large heat-evoked membrane currents with a threshold < 50 °C. Therefore, this study suggests that the MDP is a critical determinant of the heat sensitivity of both TRPV2 and TRPV1, but probably also that the as yet unidentified residues or motifs with the MDP may determine whether the channel is heat-sensitive (rodent TRPV2) or not (human TRPV2) [21]. In a more recent study from the same laboratory the authors actually refined this interpretation by identifying a small motif within the MDF that seems to dictate the thermal sensitivity of rat TRPV1 and TRPV2 [23]. By examining several chimeras with swapped fractions of the MDP from TRPV1 into TRPV2, the authors identified the residues 362–371 as a motif that encodes for the high temperature sensitivity of rat TRPV2. The chimeric TRPV2 construct containing the corresponding residues from TRPV1 displayed heat-induced currents with a threshold similar to that of TRPV1 [23]. The authors termed this motif “helix-turn-helix” because it connects two helices. Interestingly, the loop between the helices (residues 365–367) contains a deletion of a serine residue in TRPV2 compared to TRPV1. When this serine residue was inserted into the corresponding position in TRPV2 (365), the resulting mutant channel indeed exhibited heat-evoked currents with a threshold comparable to that of TRPV1 [23]. Considering that this approach was also effective on TRPV3 and TRPV4, both of which also lack this serine residue, the authors speculated that this difference between TRPV1 and TRPV2/3/4 may be due to evolutionary pressure [23]. The identified motif (362–371) in rat TRPV2 is almost perfectly conserved in human TRPV2, but it is possible relevance for the heat insensitivity of human TRPV2 was not examined. The authors finally analyzed the available data from cryo-EM structures of rat TRPV2 and determined that the motif is positioned between important regions of the channels including the S2–S3 loop, the TRP helix including the TRP domain, the proximal C-terminus and the ankyrin repeats [23]. Although the exact mechanisms that render rodent TRPV2 sensitive and human TRPV2 insensitive to heat are not yet fully understood, these two studies have identified some key mechanisms, and they clearly provide a foundation for further functional and structural studies on TRPV2. In this regard, recent reports employing cryo-EM have indeed presented the first insights into structural rearrangements of the closely related TRPV1 and TRPV3 ion channels upon exposure to heat [24, 25].

In another report from Liu and Qin, the previously mentioned use-dependency of heat-evoked membrane currents on rat TRPV2 was investigated in more detail [22]. The strong decrease in the temperature threshold of rat TRPV2 after initial activation by heat was also found in cell-free excised patches, suggesting that this effect is due to an intrinsic property of the TRPV2 proteins rather than a cytosolic regulatory mechanism [22]. They also found that the reduction in thermal threshold did not recover within 30 min. This effect appears to be irreversible, at least in a time frame that can be observed when performing patch clamp electrophysiology [22]. A similar use-dependent effect was found for the activation of rat TRPV2 by 2-APB, and an initial activation by heat can cross-sensitize the channel to 2-APB. In contrast, an initial strong activation of the channel by 2-APB did not result in a reduction of the temperature threshold for activation [22]. The authors did not yet determine the molecular mechanisms concerting this use-dependency of TRPV2, but it was suggested that it may be an important mechanism for heat sensitization associated with a thermal hyperalgesia.

We are aware of only very few reports describing TRPV2-like heat-evoked currents in dorsal root ganglion (DRG) neurons [16, 26, 27]. The main reason for this scarcity of data may be that it is technically challenging to expose DRG neurons to such high temperatures without losing the integrity of the tight seal between the cell membrane and the recording electrode used for such electrophysiological studies. Furthermore, rodent DRG neurons express several heat-sensitive ion channels, and the lack of selective TRPV2-inhibitors has made it nearly impossible to accurately study TRPV2 in these cells. Before TRPV2 was cloned, Nagy and Rang suggested the existence of a high-threshold heat sensor by demonstrating heat-evoked membrane currents with a threshold > 50 °C in medium to large sized capsaicin-insensitive rat DRG neurons [27]. Around the same time, a study by Vlachova et al. demonstrated similar high-threshold heat-evoked currents in rat DRG neurons [28]. As a logical consequence of the initial report on TRPV2 in 1999, the Nagy laboratory published data from more extensive research on such TRPV1-independent heat-evoked membrane currents in rat DRG neurons in 2002 [26]. They identified 83 medium-sized to large capsaicin-insensitive neurons generating heat-evoked currents with a mean threshold of 51.6 °C, a high permeability to calcium, a prominent use-dependency and an inhibition by the unselective TRP channel blocker ruthenium red [26]. Considering what is known about recombinant rat TRPV2 channels, these data strongly suggested that TRPV2 is likely to account for these membrane currents. This notion was supported by a study from Leffler and colleagues in 2007, demonstrating that recombinant rat TRPV2 in HEK 293 cells and capsaicin-insensitive rat DRG neurons generated high-threshold heat-evoked membrane currents with similar thresholds, use-dependency, Q_10_ values, inhibition by trivalent cations including gadolinium and lanthanum and a potentiation by 2-APB [16]. The role of TRPV2 as a sensor for high heat with a strong use-dependency was also supported by data published by Rau et al. in the same year [29]. It is noteworthy that all these studies were performed on rat DRG neurons. Considering that transgenic mice are widely used in pain research, it is surprising to note that not a single report has been published showing similar data from mouse DRG neurons. The initial study on TRPV1-knockout mice presented limited data suggesting that mouse DRG neurons could in principle generate high-threshold heat evoked currents, but these currents were not further explored [30].

With the possibility of missing reports, we came to the somewhat surprising conclusion that the last study reporting on TRPV2 and high-threshold heat-evoked currents in DRG neurons was published 17 years ago (2007) [16]. This finding probably very well reflects the general view that TRPV2 may not be a relevant molecule when it comes to studying pain.

### Expression of TRPV2 in thermosensitive sensory neurons

TRPV2 is widely expressed in different tissues including immune cells, heart, peripheral and central nervous system and in various tumors [31–37]. Obviously, thermal sensitivity with a threshold > 50 °C may not be a relevant property in most of these tissues, but at least in peripheral sensory nerves it may be. However, the ability of several substances to lower the thermal threshold for activation suggests that even modest changes of the physiological body temperature can have an impact on the activity of TRPV2 in vivo [17–19].

The expression of TRPV2 was first described in 16% of all rat DRG neurons, mainly medium-sized to large neurons most likely to be thinly myelinated Aδ fibers [2]. While co-expression with TRPV1 was not examined in this study, the authors found that 30% of all TRPV2-positive cells also contained the neuropeptide CGRP that is considered to be expressed in nociceptive sensory neurons. A similar expression pattern for TRPV2 was found in rat trigeminal ganglia, and it appears that trigeminal nerves innervating the dental pulp have a high TRPV2-expression [35, 38]. Interestingly, pulp sensory neurons are mainly nociceptors.

The previously cited report by Ahluwalia et al. used immunohistochemistry to investigate the expression of TRPV1 and TRPV2 in rat DRG neurons [26]. Essentially, these data indicated that TRPV1 and TRPV2 are expressed in distinct subpopulations with a marginal overlap. In contrast, later studies reported that up to 1/3 of all rat DRG neurons expressing TRPV2 also express TRPV1 [29, 39, 40]. Again, we find relatively little data on the expression of TRPV2 in mouse DRG neurons. Tamura et al. reported that ~ 18% of adult mouse DRG neurons express TRPV2 [41]. Park and colleagues found that ~ 16% of mouse DRG neurons showed a strong labeling for TRPV2, but that up to 34% of the may be labeled if weakly labelled cells are included [5]. Similar to rat DRG neurons, mainly medium to large sized mouse DRG neurons express TRPV2.

While all of these “early” studies may have suffered from TRPV2-antibodies of varying quality, several recent studies have analyzed the transcriptome of rodent and human DRG and trigeminal neurons. The transcriptomic results from the Price lab suggest that the strongest expression of TRPV2 in human DRG neurons is found in proprioreceptors and in the nociceptive populations referred as “pruritogen receptor enriched” and “PENK + nociceptors” [42]. While TRPV1 is not expressed in proprioreceptors, there appears to be a considerable co-expression in the two latter populations. In a cross-species transcriptomic atlas of DRG neurons from human, monkey, guinea pig and mouse, the available data for TRPV2 suggest a broad expression in most subpopulations of cells and rather diffuse species-specific differences [43]. A recent transcriptomic study of mouse trigeminal DRG neurons confirmed that neurons innervating the dental pulp express TRPV2 [44]. These are just a few examples of available transcriptomic data on sensory neurons, but so far, they seem to confirm previous studies using immunohistochemistry reasonably well. Importantly, human DRG neurons also express TRPV2 in populations similar to those found in rodent DRG neurons.

Available data on TRPV2 suggest an expression in medium to large, but also in defined populations of small DRG neurons that give rice to nociceptive unmyelinated C-fibers and thinly myelinated Aδ-fibers, as well as to proprioreceptive myelinated Aβ-fibers. Since most studies indicate that TRPV2 is highly expressed in medium to large sized neurons, early reports suggested that TRPV2 may dictate the thermosensitivity of mechano-heat-sensitive Aδ (Type 1, AMH) neurons, which have been shown to respond to a high heat with a threshold of ~ 50 °C [3, 45, 46]. As these nerves display a use-dependent response to repeated stimulations (e.g., reduced thresholds), our current knowledge of TRPV2 makes this notion to seem very likely. However, the functional validation of this hypothesis is still awaited for.

It should also be mentioned that TRPV2 is also expressed in in the spinal cord, cerebral cortex and several regions of the brain including the cerebellum, cerebrum, basal ganglia, forebrain and hypothalamus [40, 47–49]. Given that the Siemens laboratory identified a crucial role for the hypothalamus-expressed thermosensitive ion channel TRPM2 in regulating body temperature, it remains to be investigated whether TRPV2 may have similar functions in the central nervous system [50, 51].

### Lack of evidence for a physiological relevance of the thermosensitivity of TRPV2

The characterization of the role of TRPV2 in pain and thermosensitivity in in vivo models has suffered from the lack of selective agonists or antagonists. Furthermore, the classical approach of creating a genetic deletion of TRPV2 proved to be extremely challenging due to the very high perinatal lethality of mice with a global knockout of TRPV2 [5, 31]. Only 2.5% of weaned pups from heterozygous breeding pairs were fully TRPV2 knocked out available for analysis, making this the characterization of these animals difficult in many ways. Consequently, only one report has been published describing the pain phenotype of mice with global TRPV2 deficiency [5]. Effectively, the study demonstrated that mice lacking TRPV2 do not display any pain phenotype. Thus, TRPV2 knockout and wild-type mice showed more or less identical thermal and mechanical sensitivities in several models for pain [5]. Furthermore, ex vivo electrophysiological recordings from hairy or glabrous skin afferents showed normal thermal responses in C-fibers and Aδ-fibers. The authors of the study suspected that the prominent role of TRPV1 in these models might mask a less prominent role of TRPV2. Therefore, they also analyzed mice lacking both TRPV1 and TRPV2, or wildtype and TRPV2-knockout mice with an ablation of TRPV1-expressing sensory neurons with resiniferatoxin [5]. While these approaches clearly demonstrated that TRPV1 is highly important for thermosensitivity in mice, the knockout of TRPV2 did not alter the phenotype in any of the models performed. Therefore, the relevance of TRPV2 for thermosensitivity was considered to be insignificant [5].

In a more recent report, the authors analyzed mice with tissue-specific deletion of TRPV2 in cells expressing Wnt1, e.g., sensory neurons [52]. Again, the thermosensitivity was not altered in mice lacking TRPV2 in sensory afferents compared to wild-type mice. Instead, the authors reported that the in vivo and in vitro mechanical sensitivity was reduced in mice lacking TRPV2 [52]. This exciting finding indicates that TRPV2 may indeed be relevant for nociception, but rather for mechanical sensitivity than for thermal sensitivity.

We are not aware of any more recent studies that have addressed the role of TRPV2 for thermosensitivity in rodents. However, it should be noted that a recently published report demonstrated that acute thermal nociception could only be diminished a triple-knockout mice lacking TRPM3, TRPV1, and TRPA1 [53]. When only one these ion channels was expressed, the thermal nociception phenotype was more or less indistinguishable from that of wild-type mice. The authors concluded that TRPM3, TRPV1, and TRPA1 are likely to serve as redundant sensors for heat, allowing for error tolerant guidance to avoid burn injury. An independent report also demonstrated that TRPM2 expressed in sympathetic afferents, is also relevant for the detection of warmth [54, 55]. With this knowledge, the failure to detect a possible modest role of TRPV2 for thermosensitivity would likely require the deletion of multiple more prominent heat sensors.

### How is TRPV2 involved in temperature-dependent physiological functions?

Physiologically the core temperature is around 37 °C, but despite this, it has been reported that temperature is not constant in the human body. Regarding high temperatures > 50 °C, it has been reported that mitochondria can reach higher temperatures than the surrounding cell environment. While the exact physiological temperatures within mitochondria remain to be determined [56–66], the highest reported physiological temperatures in functioning mitochondria are close to 50 °C [58]. A recent study suggests that temperatures above 43 °C led to a degradation of respiratory complexes and supercomplexes in intact cells [67]. There are no studies describing a possible temperature-dependent activation of TPRV2 in mitochondria, but the expression and influence of TRPV2 on mitochondria has been discussed [68]. Despite the open question of which temperatures mitochondria may reach, the presence of reactive oxygen species could potentially lead to an activation of TRPV2 at more modest temperatures. If TRPV2 is functionally expressed in the mitochondrial membrane, this mechanism may induce to cell death.

An increase in temperature also has the potential to impact crucial functions of white blood cells, such as phagocytic activity in monocytes and superoxide production in neutrophils [69, 70]. Sensitization of rat and mouse TRPV2 by oxidants such a chloramine-T, UVA light and reactive oxygen species produced by photosensitizing agents results in a considerable decrease in the temperature threshold below body temperature (37 °C) [17]. When it comes to of human TRPV2, oxidant-induced membrane currents were enhanced by heat [17]. However, calculated Q_10_-values of this heat-induced potentiation of hTRPV2-mediated currents were > 5, indicating that elevated temperatures enhance channel activity independent of a specific mechanism [17]. Putting these properties in physiological context, phagocytosis of macrophages seems to depend on both TRPV2 and on the redox-state [17]. In addition, macrophage phagocytosis has been described to be strongly dependent on temperature [71, 72]. These properties may indicate a possible role of TRPV2 in terms of a temperature-dependent phagocytosis.

Microglia serve as the resident macrophages in the central nervous system. In their resting state they monitor environmental changes, while in an activated state they take protective measures to restore equilibrium [73, 74]. Recently, it was shown that TPRV2 is expressed in microglia and that nitric oxide upregulates microglial phagocytosis and increases TRPV2 expression on the plasma membrane [75, 76]. Microglial movement has been shown to be temperature-dependent both in vitro and in vivo. Studies on genetically modified mice and pharmacological tools revealed that the temperature-dependent movement of microglia primarily depend on the activity of TRPV4 [77]. The potential role of TRPV2 in these processes has not yet been investigated.

## Conclusion and future perspectives

In this topical review, we describe the currently available data on the properties and significance of the thermosensitivity of TRPV2. Although the amount of data is rather limited when compared to other thermosensitive TRP channels such as TRPV1 and TRPA1, there is solid evidence that at least rodent TRPV2 exhibits a strong thermosensitivity both as recombinant proteins and as endogenously expressed ion channels in neurons. The high threshold for activation exceeding 50 °C may not be a highly relevant physiological trigger for TRPV2-activity, but it is easy to imagine that the ability of several other modulators of TRPV2 to reduce this threshold is likely to be of physiological relevance. So far, the efforts to gain more insight into this property of TRPV2 have failed to identify a significant role in mice. There seems to be more “room” elaborate this further, but the scientific community has not yet prioritized pursuing this open question. A main reason for this lack of interest is probably the heat-insensitivity of human TRPV2, which makes the translational perspective of relevance to human physiology and pathophysiology rather unlikely. Nevertheless, a broad physiological relevance has meanwhile been described for TRPV2. We suggest that the combination of classical physiological approaches with novel techniques like cryo electron microscopy (cryo-EM), human induced pluripotent stem cells and “omics” has great potential to provide us with a more complete understanding of how, when and why TRPV2 may be relevant for thermosensitivity.

## Data Availability

Not applicable.

## Abbreviations

TRP channel: Transient receptor potential channel
DRG: Dorsal root ganglion
HEK Cell 293: Human embryonic kidney cell 293
Cryo-EM: Cryo electron microscopy
MDP: Membrane-proximal domain
